# A Systemically-Administered Small Molecule Antagonist of CCR9 Acts as a Tissue-Selective Inhibitor of Lymphocyte Trafficking

**DOI:** 10.1371/journal.pone.0050498

**Published:** 2012-11-29

**Authors:** Noah J. Tubo, Marc A. Wurbel, Trevor T. Charvat, Thomas J. Schall, Matthew J. Walters, James J. Campbell

**Affiliations:** 1 Brigham and Women’s Hospital, Department of Dermatology, Boston, Massachusetts, United States of America; 2 Harvard Medical School, Program in Biological and Biomedical Sciences, Boston, Massachusetts, United States of America; 3 ChemoCentryx, Inc., 850 Maude Avenue, Mountain View, California, United States of America; 4 Harvard Medical School, Departments of Dermatology and Pathology, Boston, Massachusetts, United States of America; University of California, San Francisco, United States of America

## Abstract

A goal for developers of immunomodulatory drugs has long been a systemically administered small molecule that can selectively inhibit inflammation in specific tissues. The chemokine receptor CCR9 is an attractive target for this approach, as entry of T cells into the small intestine from blood requires interaction between CCR9 and its ligand CCL25. We have tested the ability of a small molecule CCR9 antagonist, CCX8037, to inhibit antigen-mediated T cell accumulation in the intestine. This compound prevented accumulation of gut-imprinted antigen-specific CD8 T cells within epithelium of the small intestine. Interestingly, the antagonist did not affect the robust generation of gut-imprinted CD8 T cells within mesenteric lymph nodes. To distinguish “gut-selective” from “general” T cell inhibition, we tested the drug’s ability to influence accumulation of T cells within skin, a tissue in which CCR9 plays no known role, and we found no appreciable effect. This study demonstrates the feasibility of creating systemically-administered pharmaceuticals capable of tissue-selective immune modulation. This proof of concept is of utmost importance for designing effective treatments against various autoimmune disorders localized to a specific tissue.

## Introduction

The circulating T cell pool contains multiple antigen-experienced subsets bearing distinct tissue tropisms. The two best understood are those associated with skin and intestine. Together, these two populations comprise at least half of all blood-borne Ag-experienced T cells [1]. Each subset is responsible for immunological memory and immunosurveillance of its own target tissue. In both mice and humans, skin-homing cells express E-selectin ligand (E-lig) and chemokine receptor 4 (CCR4), while small-intestine-homing cells express integrin α4β7 and CCR9 [1]. Each of these molecules is required for normal homing of each cell type to its respective target organ [2].

Currently available immunosuppressants tend to immunocompromise patients overall, leaving them susceptible to opportunistic infection within any given tissue. In contrast, a tissue-selective immunomodulatory agent might ameliorate lesions in the affected site without rendering immunologically healthy tissues vulnerable to infection. As such, the lymphocyte trafficking field has long held as its “holy grail” the notion that a systemically administered pharmaceutical might be designed to selectively attenuate localized autoimmune symptoms [2]. There is precedent that blocking the function of homing molecules can affect inflammation quite dramatically. For example, natalizumab, a humanized monoclonal antibody against the α4 integrin chain, is FDA approved as an anti-inflammatory agent (reviewed in [3]). However, the targeted integrin chain is a component of several distinct integrin heterodimers, and is not associated with selective lymphocyte trafficking to any specific tissue [2].

Nonetheless, drugs intended to modulate selective homing of T cells to particular tissues have not been as uniformly successful as previously hoped [4]. The disappointing outcome of this approach to date may be partially explained by the discovery that many tissue-selective homing mechanisms rely on competition among lymphocyte subsets for entry into tissue from the circulation. For example, normal T cells are 20-fold more likely to accumulate within inflamed skin than otherwise identical cells that lack CCR4 [5], [6], [7]. However, CCR4^−/−^ T cells do gain access to skin when such competition is removed; CCR4^−/−^ mice have relatively normal densities of T cells in both inflamed and resting skin [8]. Thus, CCR4 is required for skin homing only in a physiologically competitive environment. Ablation of the CCR4 function alters the environment such that CCR4 is no longer needed for skin homing. Less efficient (perhaps even non-physiological) mechanisms may then take over in guiding lymphocytes into tissues.

Homing of T cells to the intestine appears to provide a more promising target for tissue-selective pharmaceutical manipulation. Humanized antibodies to the integrin heterodimer α4β7 (vedolizumab) or its ligand MAdCAM-1 (PF-00547,659) have provided clinical improvements in ulcerative colitis and Crohn’s disease in Phase I and II trials (reviewed in [3]).

A small molecule antagonist of CCR9 recently demonstrated efficacy in the PROTECT-1 clinical trial for Crohn’s disease (reviewed in [9], [10], [11]). The ability of an antagonist of this nature to modulate a local immune response within a tissue, after systemic dosing, while allowing normal immune function in other tissues has not been described previously. We therefore tested a murine-optimized version of this drug in mouse models of skin and gut inflammation to assess its relative efficacy in cutaneous versus intestinal inflammation.

We found that the inhibition of CCR9 function with a specific antagonist is extremely effective at excluding Ag-specific inflammatory CD8 T cells from intestinal epithelium, without impacting the recruitment of antigen-specific cells to the inflamed skin. To our knowledge this is the first direct evidence that a systemically administered small molecule can effectively treat inflammation in a tissue-selective manner.

## Results and Discussion

### CCX8037 is a Potent and Selective CCR9 Antagonist

**Figure 1:**
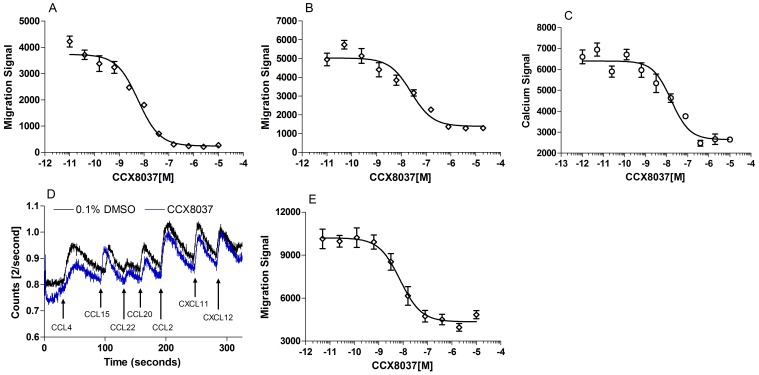
CCX8037 is a potent and selective antagonist of CCR9. CCL25-induced chemotaxis was measured on Molt-4 cells and murine thymocytes by using a DNA intercalating fluorescent dye (CyQUANT) to quantify responding cells and is labeled as the “migration signal”, as the relative fluorescence of the migrated population is proportional to the number of cells that migrated. Six to eight replicates were performed per data point. Calcium flux was measured on Molt-4 cells or IL-2-cultured lymphocytes loaded with Indo-1AM dye and exposed to IC_50_ concentrations of the chemokines indicated. A) CCX8037 inhibits CCL25-induced Molt-4 chemotaxis in buffer (0.1% BSA in HBSS) with an IC_50_ of 12 nM (n = 31). B) CCX8037 inhibits CCL25-induced Molt-4 chemotaxis in the presence of 100% human AB serum with an IC_50_ of 32 nM (n = 9). C) CCX8037 inhibits CCL25-mediated mobilization of intracellular Ca2+ in Molt-4 cells with an IC_50_ of 19 nM (n = 3). D) CCX8037 (blue trace; 10 uM) does not inhibit chemokine induced mobilization of intracellular Ca2+ to EC_50_ concentrations of CCL4(CCR5), CCL15 (CCR1) on IL-2 activated lymphocytes. E) CCX8037 inhibits CCL25-induced murine thymocyte chemotaxis in buffer with an IC_50_ of 2.5 nM (n = 2).

CCR9 dependent chemotaxis can be readily assessed *in vitro* using the Molt-4 T cell line, which endogenously expresses CCR9 and responds to CCL25 with a stereotypical bell-shaped chemotaxis curve in standard *in vitro* chemotaxis assays [11]. CCX8037 is a potent inhibitor of CCL25-mediated Molt-4 chemotaxis in buffer (0.1% BSA in HBSS) with an IC_50_ of 12 nM (Fig. 1A). In order to assess the potency of this molecule under physiologically relevant conditions, chemotaxis assays were performed in the presence of 100% human AB serum: CCX8037 inhibited CCL25-induced Molt-4 chemotaxis with an IC_50_ of 32 nM under these conditions (Fig. 1B). In addition to inhibiting CCL25-induced chemotaxis, CCX8037 also inhibits CCL25-induced Ca^2+^ mobilization in Molt-4 cells with an IC_50_ of 19 nM (Fig. 1C) To determine its potency against mouse CCR9, a murine thymocyte chemotaxis assay was performed. CCX8037 inhibited CCL25-induced chemotaxis of murine thymocytes with an IC_50_ of 2.5 nM (Fig. 1E).

The selectivity of CCX8037 for CCR9 was assessed in real time using IL-2 cultured lymphocytes that were stimulated sequentially with the indicated chemokines (Fig. 1D) in the presence of either 0.1% DMSO or 10 µM of compound. Cells stimulated in the presence of CCX8037 did not exhibit any appreciable reduction in chemokine induced Ca^2+^ mobilization relative to the DMSO control for any of the non-CCL25 chemokines tested.

### CCR9 Antagonist Inhibits Homing of OT-I CD8 T Cells to the Intestinal Epithelium after Oral Immunization

To evaluate the effectiveness of CCX8037 at inhibiting CCR9-mediated chemotaxis and trafficking *in vivo*, we modified a model system that we employed previously to study the role of CCL25 in homing of CD8 T cells to the small intestinal epithelium (IE) [12]. Congenically marked CD45.1^+^ CD8 T cells from TCR-transgenic OT-I mice (specific for ovalbumin peptide OVA_257-264_ in the context of H-2K^b^) [13] were adoptively transferred into WT CD45.2^+^ mice. One day following adoptive transfer, mice were administered adjuvant (cholera toxin (CT)) by oral gavage, with or without antigen (OVA). Those mice that received both adjuvant and antigen were split into two treatment groups. Each group received subcutaneous injections of either saline alone (control group) or 30 mg/kg CCX8037 in saline (experimental group) every 12 hours. The dose of CCX8037 administered to the experimental group was designed to sustain plasma concentrations at or above the serum adjusted IC_90_ of 320 nM during the 12 hours between injections (The IC_90_ represents the concentration of CCX8037 that is required to block 90% of the CCL25-induced chemotaxis response). This regimen was maintained for 5 days post immunization, after which the mice were sacrificed and lymphocyte cell suspensions were prepared from MLN and intestinal epithelium (IE). Blood samples were taken at the time of sacrifice to confirm that drug levels were at the IC_90_ of 320 nM (data not shown). [Note: oral gavage is often used as a technique for generating immune tolerance. This is not relevant in this case, as the CT adjuvant effectively breaks induction of tolerance [12]].

**Figure 2:**
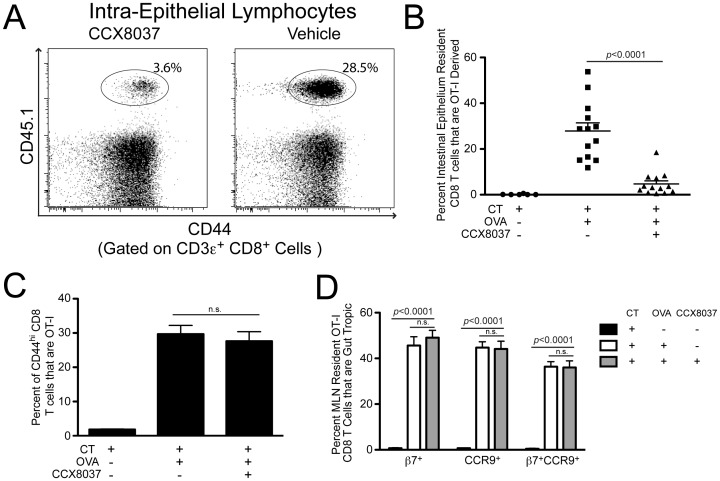
CCX8037 reduces accumulation of OT-I CD8 T cells in the intestinal epithelium without affecting gut homing tropism, imprinting and proliferation. Animals were injected with 3e6 OT-I CD8 T cells, and immunized 24 hours via oral gavage with either 10 µg Cholera Toxin (CT) only, or CT + 25 mg Ovalbumin (OVA). Animals given OVA were also injected subcut. every 12 hours for the course of the study with CCX8037 (30 mg/kg) or vehicle. Mice were sacrificed for analysis 5 days post immunization. Mean and SEM shown for each data point, *p* values indicate Bonferroni multiple comparison post test. (A) Representative flow cytometry plot showing the accumulation of CD44^hi^ CD8 T cells, and gating of OT-I (CD45.1) cells in the intestinal epithelium. Plots are pre-gated on CD3ε^+^/CD8α^+^ cells. (B) Quantification of OT-I CD8 T cell accumulation in intestinal epithelium. Mice fed CT only did not exhibit substantial OT-I CD8 T cell homing into the intestinal epithelium. Animals fed CT + OVA and treated with vehicle had significant OT-I CD8 T cell homing, with 27.9% of all resident CD8 T cells being OT-I derived. Animals fed CT + OVA and injected with CCX8037 exhibited significantly reduced intestinal epithelium accumulation of OT-I CD8 T cells, to 4.7%. N = 13 mice for CCX8037 and vehicle groups, and 6 for CT only. (C) CCX8037 did not affect the proliferation of OT-I CD8 T cells in MLN after Ag exposure. In animals exposed to CT only, OT-I CD8 T cells composed 1.8% of all CD44^hi^ CD8 T cells. In animals exposed to CT + OVA, there was no significant difference in the percentage of CD44^hi^ CD8 T cells that are OT-I between those treated with vehicle (29.7%) and CCX8037 (27.6%). N = 6 for CT only treated mice, N = 13 for Vehicle and CCX8037 treated mice. (D) Generation of gut homing molecules on OT-I CD8 T cells in MLN was not affected by CCX8037. Animals not fed OVA antigen had significantly lower β7^+^, CCR9^+^, or β7^+^CCR9^+^ expression. However, the expression of gut homing molecules was not significantly affected by CCX8037 treatment, compared to vehicle. OT-I CD8 T cells of vehicle and CCX8037 treated mice were 45.6% and 49.1% β7^+^ respectively, 44.7% and 44.1% CCR9^+^ respectively, and 36.3% and 36.1% β7^+^CCR9^+^ respectively. N = 13 mice for CCX8037 and vehicle groups, and 6 for CT only.

We found the proportion of donor-derived OT-I cells within the total CD8 T cell population of IE to be reduced by ∼6-fold in mice treated with CCX8037 (Fig. 2A and 2B). Interestingly, this reduction was similar in magnitude to that seen for OT-I cells transferred to mice congenitally lacking CCL25 [12]. The number of OT-I CD8 T cells found in the IE was negligible in mice that received only adjuvant (Fig. 2B).

The reduction of OT-I cells within the total CD8 T cell intestinal epithelium population after treatment does not by itself imply the mechanism of action. The CCR9 antagonist may indeed interfere directly with CCR9-mediated homing to intestinal epithelium. However, the reduction could be explained equally well by effects of CCX8037 within the GALT, acting to reduce Ag-induced OT-I proliferation, or to reduce differentiation of naïve precursors into gut-selective memory or effector populations that express intestinal homing markers.

To distinguish among these possibilities, we analyzed OT-I cells from a representative GALT tissue, the mesenteric lymph node (MLN), to assess their proliferation and gut-selective differentiation status. We used CD44 as a marker to distinguish naïve cells (CD44^lo^) from those that had previously responded to cognate Ag (CD44^hi^). In the absence of Ag, OT-I cells comprised only ∼2% of the total CD44^hi^ CD8 T cell population in MLN (Fig. 2C). After immunization, OT-I cells increased to ∼30% of the total CD44^hi^ CD8 T cell population. Importantly, the presence of CCX8037 did not appreciably influence Ag-specific proliferation in this assay (Fig. 2C).

**Figure 3:**
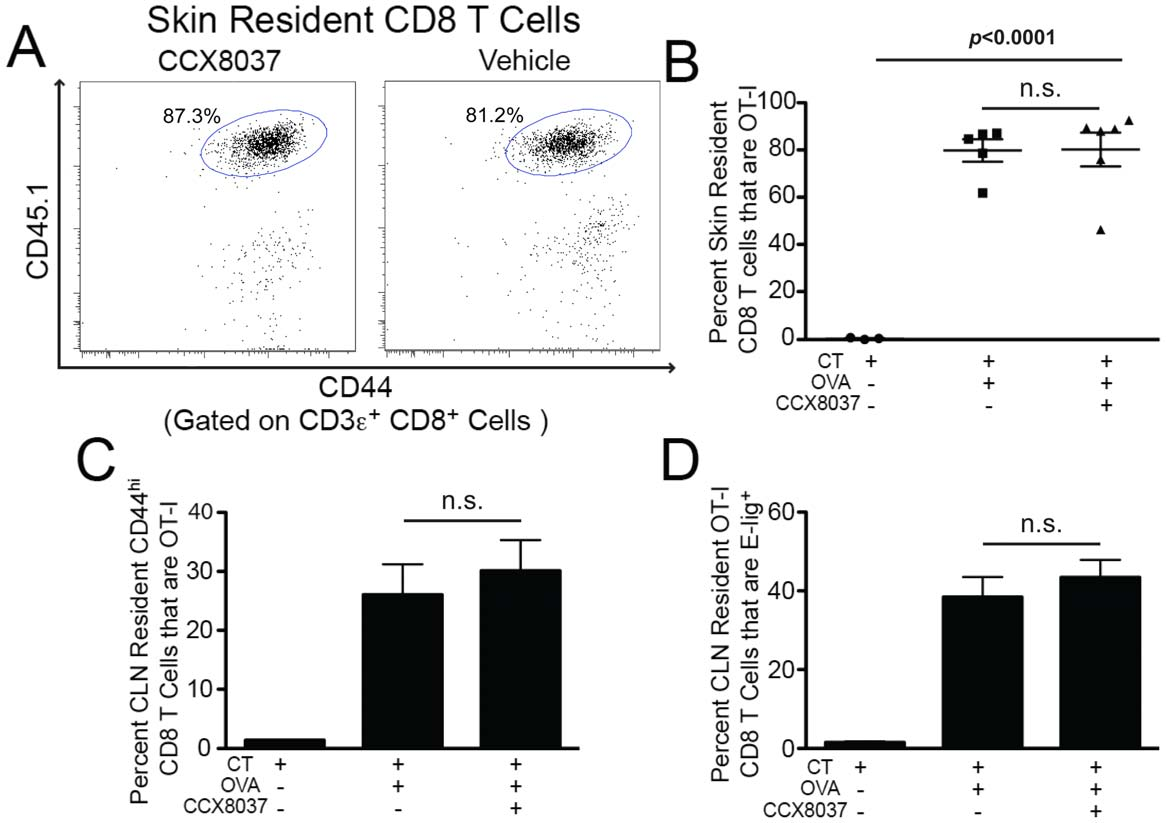
CCX8037 does NOT reduce accumulation of OT-I CD8 T cells in the skin. Animals were injected with 3e6 OT-I CD8 T cells, and epicutaneously immunized 24 hours later on the ear skin. Ear skin was painted with either 100 µg Cholera Toxin (CT) only, or 100 µg CT + 100 µg OVA_257–264_. Animals treated with OVA_257–264_ were split into two groups and received subcut. injections of CCX8037 or vehicle every 12 hours for the course of the study. Mice were sacrificed for analysis 5 days post immunization. Mean and SEM shown for each data point, *p* values indicate Bonferroni multiple comparison post test. (A) Representative flow cytometry plot showing the accumulation of CD44^hi^ CD8 T cells, and gating of OT-I (CD45.1) cells in the ear skin. Plots are pre-gated on CD3ε^+^ CD8α^+^ cells. (B) Quantification of OT-I CD8 T cell accumulation in ear skin. Mice treated with CT only did not exhibit substantial OT-I CD8 T cell homing into the ear skin. Mice treated CT + OVA_257-264_ and treated with vehicle had significant OT-I CD8 T cell homing, with 79.6% of all resident CD8 T cells being OT-I derived, while those treated with CCX8037 had 80.2% of all CD8 T cells being OT-I derived. N = 5 groups for vehicle, 6 for CCX8037 and 3 for CT only. Each group was comprised of 3–5 pooled mice. (C) CCX8037 did not affect the proliferation of OT-I CD8 T cells in CLN after Ag exposure. In animals exposed to CT only, OT-I CD8 T cells composed 1.4% of all CD44^hi^ CD8 T cells. In animals exposed to CT + OVA, there was no significant difference in the percentage of CD44^hi^ CD8 T cells that are OT-I between those treated with vehicle (26.0%) and CCX8037 (30.1%). N = 3 groups for CT only treated mice, N = 6 groups for Vehicle and CCX8037 treated mice, where each group is 3–5 pooled mice. (D) Generation of skin homing associated molecule E-selectin ligand by OT-I CD8 T cells in CLN was not affected by CCX8037. When mice were immunized with OVA antigen, the E-lig was not significantly affected by the presence of the CCX8037 (38.4% vehicle, 43.3% CCX8037). In the absence of OVA antigen, E-lig production by OT-I CD8 T cells was negligible (<1% E-lig^+^). N = 4 for CT only, 16 for Vehicle and CCX8037.

We next examined the Ag-induced expression of gut-tropic homing molecules (*i.e*. tissue-specific imprinting) [14] on OT-I cells within the MLN. We assessed expression of β7-integrin and CCR9 by flow cytometry. Oral immunization with OVA caused dramatic increases in β7 and CCR9 expression, but CCX8037 did not appreciably affect expression of either molecule alone or in combination (Fig. 2D). These homing molecules were rarely present on OT-I cells in the mice immunized orally with adjuvant only. Thus, a reduction in gut-specific Ag-induced imprinting within GALT does not explain the CCX8037-induced reduction of OT-I cells within the intraepithelial CD8 T cell pool.

Taken together, these data strongly suggest that the effects mediated by CCX8037 in intestinal epithelium are due to direct interference with the trafficking of Ag-specific T cells into the tissue.

### Extravasation of OT-I CD8 T Cells to Inflamed Skin is Unaffected by CCR9 Antagonist

The prior experiments did not rule out the possibility that CCX8037 caused a general defect in trafficking of CD8 T cells to all peripheral tissues. To see if this was the case, we tested the ability of CCX8037 to inhibit Ag-specific accumulation of OT-I cells within the skin [1]. OT-I CD8 T cells were adoptively transferred into WT CD45-congenic recipients exactly as performed in the gut-homing studies above. Animals were immunized topically on the ear skin with either CT only or with CT + OVA. As above, animals that received both antigen and adjuvant were split into two groups for injection of CCX8037 or vehicle every 12 hours. Mice were sacrificed 5 days post immunization and cell suspensions were prepared directly from the inflamed ear skin and from the cervical lymph nodes (cLN), which are the lymphoid organs that directly drain the site of inflammation.

In striking contrast to the findings in gut, CCX8037 did not lead to any appreciable reduction in the proportion of CD8 T cells within skin that were derived from the OT-I donor (Figs. 3A and B). Ag-induced proliferation of OT-I cells within the cLN was not affected by CCX8037, nor was their imprinting with the skin-homing molecule E-lig (Fig. 3C and 3D).

Unlike gut-selective homing of CD8 T cells, skin specific homing does not depend upon CCR9 function. Having shown that CCX8037 compromises CD8 T cell homing to the gut, but not to the skin *in vivo*, we find that a small molecule drug can be administered systemically to selectively inhibit CCR9-mediated trafficking of CD8 T cells to the small intestine.

### Concluding Remarks

In this study, we have demonstrated *in vivo* that a small molecule inhibitor of CCR9 can prevent T cell accumulation within inflamed intestine without affecting inflamed skin. We believe this to be the first clear-cut *in vivo* example of a small molecule able to modulate tissue-selective T cell mediated immunity, a strategy that may well be used to modulate autoimmune syndromes in the intestine, as well as autoimmune syndromes in other tissues.

## Materials and Methods

### Ethics Statement

All experimental procedures involving animals were approved by the Children’s Hospital Boston (CHB) IACUC (*protocol number 10-04-1645R*), which oversees the facility in which these procedures were performed.

### Mice

C57BL/6 n mice (CD45.2^+^) were purchased from Charles River Labs (Wilmington, MA). OT-I Tg mice were bred onto the CD45.1 C57BL/6 n background ((B6.SJL-*Ptprca Pep3b*/BoyJ; The Jackson Laboratory). Animals were housed under SPF conditions.

### Cell Culture, Chemotaxis and Mountain Peak Assays

Molt-4 cells were obtained from the ATCC (Manassas, VA) and cultured in RPMI-1640 (Invitrogen, Carlsbad, CA) supplemented with 10% FCS (Invitrogen, Carlsbad, CA) in a humidified 5% CO_2_ incubator at 37°C. Chemotaxis assays were conducted as previously described [11], responding cells were quantified by the addition of CyQUANT (Invitrogen) to the cells and measuring the resulting fluorescence using a Spectraflour Plus plate reader (Tecan, Grodig, Austria). Mouse thymocytes for determining the murine potency of CCX8037 were isolated from 3–6 week old C57BL/6 mice (The Jackson Laboratory, Sacramento CA). Mountain peak assays were performed using IL-2 cultured lymphocytes as previously described [11].

### Compound

CCX8037 was provided by Dr. J. Powers, Medicinal Chemistry Department , ChemoCentryx (Mountain View, CA).

### Adoptive Transfers and Immunizations

Adoptive transfers were performed as described previously[12]. Briefly, single cell suspensions were prepared from spleens and lymph nodes of OT-I Tg CD45.1 mice. 3×10^6^ CD45.1 CD8 T cells were injected retro-orbitally into sex-matched congenic CD45.2 C57BL/6 n mice (8–10 weeks old). 24 hours later, animals were either immunized via oral gavage with 5 mg Ovalbumin protein (Sigma-Aldrich, St. Louis, MO) + 10 µg Cholera Toxin (Calbiochem, San Diego, CA), or epicutaneously on the ear skin with 300 µg OVA_257–264_ peptide (Biomatik, Ontario, Canada) and 100 µg Cholera toxin by ear painting after tape stripping and acetone treatment as described previously[6].

### Isolation of Lymphocytes from Intestinal Epithelium

IEL from the small intestine were isolated as described: Peyer’s Patches were removed and, after flushing with PBS, the gut was opened longitudinally and laterally into 0.5-cm pieces. The small intestinal mucosa was then dissociated by stirring in 25 ml of medium containing 0.5% FCS, 10 mM EDTA and 1 mM DTET for 2 times 20 min at 37°C. IEL were further purified on a 40%–80% Percoll gradient.

### Isolation of CD8 T Cells from Skin

Lymphocytes were isolated from skin as follows: ears were harvested from mice, and dorsal and ventral surfaces were split apart with forceps. Ear halves were diced into ∼0.5 mm pieces. Ear pieces were incubated in HBSS + 2 mM EDTA + 10 mM HEPES for 4 hours at 4°C with constant brisk stirring. Ear solution was passed through a 40 µM filter, and cells were centrifuged from the suspension, and washed 2X with PBS + 10% bovine Serum.

### Flow Cytometry

All flow cytometry was performed on a BD FACS Canto flow cytometer. Antibodies and staining reagents were obtained from eBioscience (San Diego, CA), BD Pharmingen (San Diego, CA), R&D systems (Minneapolis, MN), and Jackson Immunoresearch (West Grove, PA). Analysis of flow cytometry data was performed using Treestar FlowJo v.8.8.2 (Ashland, OR), and Graphpad Prism v.5.0a (La Jolla, CA).

## References

[1] Campbell JJ, Butcher EC (2000) Chemokines in tissue-specific and microenvironment-specific lymphocyte homing. Curr. Opin. Immunol. 12: 336–341. 10.1016/s0952-7915(00)00096-0 10781407

[2] Butcher EC, Williams M, Youngman K, Rott L, Briskin M (1999) Lymphocyte trafficking and regional immunity. Adv. Immunol. 72: 209–253. 10.1016/s0065-2776(08)60022-x 10361577

[3] Villablanca EJ, Cassani B, von Andrian UH, Mora JR (2011) Blocking lymphocyte localization to the gastrointestinal mucosa as a therapeutic strategy for inflammatory bowel diseases. Gastroenterology 140: 1776–1784. 21530744 10.1053/j.gastro.2011.02.015 PMC3102304

[4] Zollner TM, Asadullah K, Schon MP (2007) Targeting leukocyte trafficking to inflamed skin: still an attractive therapeutic approach? Exp. Dermatol. 16: 1–12. 10.1111/j.1600-0625.2006.00503.x 17181631

[5] Baekkevold ES, Wurbel MA, Kivisakk P, Wain CM, Power CA, et al (2005) A role for CCR4 in development of mature circulating cutaneous T helper memory cell populations. J. Exp. Med. 201: 1045–1051. 10.1084/jem.20041059 PMC2213118 15795234

[6] Campbell JJ, O’Connell DJ, Wurbel MA (2007) Cutting Edge: Chemokine receptor CCR4 is necessary for antigen-driven cutaneous accumulation of CD4 T cells under physiological conditions. J Immunol. 178: 3358–3362. 10.4049/jimmunol.178.6.3358 PMC2575766 17339428

[7] Tubo NJ, McLachlan JB, Campbell JJ (2011) Chemokine receptor requirements for epidermal T-cell trafficking. Am. J. of Pathol. 178: 2496–2503. 10.1016/j.ajpath.2011.02.031 PMC3124236 21641376

[8] Reiss Y, Proudfoot AE, Power CA, Campbell JJ, Butcher EC (2001) CC chemokine receptor (CCR)4 and the CCR10 ligand cutaneous T cell-attracting chemokine (CTACK) in lymphocyte trafficking to inflamed skin. J. Exp. Med. 194: 1541–1547. 10.1084/jem.194.10.1541 PMC2193675 11714760

[9] Eksteen B, Adams DH (2010) GSK-1605786, a selective small-molecule antagonist of the CCR9 chemokine receptor for the treatment of Crohn’s disease. IDrugs 13: 472–781. 20582872

[10] Keshav S, Johnson D, Bekker P, Schall TJ (2009) PROTECT-1 Study Demonstrated Efficacy of the Intestine-Specific Chemokine Receptor Antagonist CCX282-B (Traficet-EN) in Treatment of Patients with Moderate to Severe Crohn’s Disease. Gastroenterology 136: A65–A65.

[11] Walters MJ, Wang Y, Lai N, Baumgart T, Zhao BN, et al (2010) Characterization of CCX282-B, an orally bioavailable antagonist of the CCR9 chemokine receptor, for treatment of inflammatory bowel disease. J. Pharmacol. Exp. Ther. 335: 61–69. 10.1124/jpet.110.169714 20660125

[12] Wurbel MA, Malissen M, Guy-Grand D, Malissen B, Campbell JJ (2007) Impaired accumulation of antigen-specific CD8 lymphocytes in chemokine CCL25-deficient intestinal epithelium and lamina propria. J. Immunol. 178: 7598–7606. 10.4049/jimmunol.178.12.7598 PMC2564614 17548595

[13] Hogquist KA, Jameson SC, Heath WR, Howard JL, Bevan MJ, et al (1994) T cell receptor antagonist peptides induce positive selection. Cell 76: 17–27. 8287475 10.1016/0092-8674(94)90169-4

[14] Sigmundsdottir H, Butcher EC (2008) Environmental cues, dendritic cells and the programming of tissue-selective lymphocyte trafficking. Nat. Immunol. 9: 981–987. 10.1038/ni.f.208 PMC3171274 18711435

